# Dental Caries

**Published:** 1884-12

**Authors:** 


DENTAL CARIES.—A critical summary on the Prevention of Dental Caries, By Henry Sewell, M. R. C. S., L. D. S., England. A series of papers reprinted from the Journal of the British Dental Association.

These papers present a well written essay upon the Predisposing and Exciting or Immediate Causes of Dental Caries, and are well worthy of careful perusal.

That Dr. Sewell's theories concerning the causes of dental caries, as they are presented in this volume, are correct, will be the verdict of the majority of the profession who will peruse its pages, and much that is instruction may be gathered from them.

